# Modulating NPC1L1 to Potentiate PARP Inhibitor-Induced Ferroptosis and Immune Response in Triple-Negative Breast Cancer

**DOI:** 10.3390/pharmaceutics17050554

**Published:** 2025-04-24

**Authors:** Ge Li, Yuxia Yuan, Xinhua Wu, Lixian Wu

**Affiliations:** 1 Department of Pharmacology, School of Pharmacy, Fujian Medical University, Fuzhou 350100, China; glorialee2022@126.com (G.L.); yyx19960829@163.com (Y.Y.); 13559354655@163.com (X.W.); 2 Fujian Key Laboratory of Natural Medicine Pharmacology, Fujian Medical University, Fuzhou 350100, China

**Keywords:** PARP inhibitor, lipid metabolism, NPC1L1, AKT inhibitor, immune response

## Abstract

**Background/Objectives:** Poly (ADP-ribose) polymerase (PARP) inhibitors have shown significant efficacy in treating BRCA-mutated cancers; however, a significant proportion of patients fail to respond. Emerging evidence highlights the role of PARP in lipid metabolism, suggest-ing its modulation as a novel strategy to regulate tumor progression. **Methods:** In this study, lipidomics and transcriptomics analyses were conducted to elucidate the mechanisms underlying PARP inhibitor-induced ferroptosis and immune modulation in triple-negative breast cancer (TNBC). **Results:** We demonstrated that the PARP inhibitor Niraparib significantly reprograms lipid metabolism in TNBC cells, marked by elevated phosphatidylethanolamine (PE) and cholesterol ester (ChE) levels. This metabolic shift was mechanistically linked to upregulation of the cholesterol transporter NPC1L1 via the PARP1-RELA-NPC1L1 signaling axis, which subsequently activated the AKT pathway. Combinatorial treatment with Niraparib and either Ezetimibe (an NPC1L1 inhibitor) or AZD5363 (an AKT inhibitor) synergistically enhanced TNBC cell death by promoting ferroptosis through glutathione depletion and lipid peroxidation. Furthermore, NPC1L1 inhibition amplified PARP inhibitor-induced immune responses, increasing CD8^+^ T cell infiltration and cytotoxicity in tumors. **Conclusions:** In conclusion, our findings establish NPC1L1 as a critical mediator of PARP inhibitor efficacy and propose dual targeting of lipid metabolism, providing a new therapeutic approach for the combination treatment of TNBC.

## 1. Introduction

In 2005, two landmark studies demonstrated that PARP inhibitors exhibit selective cytotoxicity in cancer cells harboring BRCA1/2 mutations or displaying homologous recombination deficiency (HRD) [1,2]. These findings have led to the development and clinical approval of four different PARP inhibitors for the treatment of a variety of malignant tumors. Notably, Niraparib, a potent PARP inhibitor, was authorized for advanced ovarian cancer regardless of homologous recombination (HR, a high-fidelity DNA repair mechanism) status, underscoring its broad therapeutic potential [3,4]. Despite their clinical success, PARP inhibitors exhibit notable limitations, including suboptimal efficacy in subsets of BRCA-mutated cancers and the inevitable emergence of resistance mechanisms [5,6]. To overcome these challenges, recent investigations have focused on non-canonical roles of PARP proteins, such as their involvement in metabolic reprogramming and innate immune regulation. In addition, rational combination therapies have been developed. These efforts are aimed at increasing therapeutic efficacy, overcoming resistance and expanding the applicability of PARP inhibitors beyond genetically defined populations.

The PARP protein family, traditionally associated with DNA repair processes, has recently emerged as a critical regulator of lipid metabolism [7]. These enzymes are not only influenced by lipid-derived metabolites but also engage in crosstalk with transcription factors governing lipid homeostasis [7,8]. A hallmark of cancer metabolic reprogramming is the preferential reliance on de novo lipogenesis over exogenous lipid uptake or hepatic synthesis. This divergence from normal cellular physiology maintains membrane biogenesis and redox balance in proliferating tumors [9]. Given the multifaceted relationship between PARP proteins and lipid metabolism, the potential of PARP inhibition to regulate lipid-related tumor suppression is an intriguing area for further research.

Recent advances in cancer metabolism research have elucidated diverse mechanisms by which lipid metabolic reprogramming sustains tumor growth and survival. A pivotal discovery is ferroptosis, a type of cell death caused by excessive lipid peroxidation, which does not depend on traditional cellular energy pathways [10]. Mechanistically, the process of ferroptosis is distinguished by the collapse of the glutathione (GSH)-dependent antioxidant system, iron overload, and peroxidation of polyunsaturated fatty acid (PUFA)-containing phospholipids, which ultimately lead to membrane rupture. In order to evade this lethal process, tumor cells employ adaptive strategies, such as the downregulation of PUFA incorporation into membranes, thereby reducing their susceptibility to lipid peroxidation [11]. Since its formal definition in 2012, ferroptosis has become a significant area of study [12]. It has been demonstrated that elevated levels of cholesterol in breast cancer cells require the sustained expression of GPX4, a negative regulator of ferroptotic cell death. This has been shown to contribute to cancer progression and its subsequent spread [13].

Cholesterol plays a complex role in ferroptosis. It directly inhibits lipid peroxidation through its antioxidant effects, with 7-dehydrocholesterol (7-DHC), a precursor of cholesterol, being particularly effective at inhibiting ferroptosis. Specifically, 7-DHC stabilizes membrane phospholipids by shielding them from autoxidation-driven fragmentation, thereby preserving membrane integrity and mitigating oxidative damage [14,15]. Although 7-dehydrocholesterol reductase (DHCR7) activity promotes ferroptosis by depleting the anti-ferroptotic metabolite 7-DHC, DHCR7 deficiency strongly suppressed ferroptosis induced by RSL3 or ML210 [16]. It has been demonstrated that cholesterol orchestrates metabolic reprogramming in the tumor microenvironment (TME) by means of upregulating CD36 expression. CD36, a fatty acid translocase, enhances lipid uptake in tumor-infiltrating CD8^+^ T cells, which has the potential to drive excessive lipid peroxidation and ferroptosis [17,18]. Moreover, alterations in cholesterol levels due to dysregulated squalene biosynthesis have been demonstrated to affect cellular redox balance and ferroptosis sensitivity. Squalene accumulation exerts a cytoprotective effect by attenuating lipid peroxidation (LPO) and suppressing ferroptotic signaling cascades [19].

NPC1L1, also known as Niemann-Pick C1-Like 1, is a pivotal cholesterol transport protein. It is primarily expressed in the small intestine and liver, where it plays a crucial role in regulating systemic cholesterol homeostasis. By mediating the absorption of dietary cholesterol from the intestinal lumen into the bloodstream and its subsequent transport to hepatic and adipose tissues, NPC1L1 ensures precise regulation of cholesterol balance [20]. The role of NPC1L1 in triple-negative breast cancer (TNBC) is increasingly recognized, with studies suggesting its involvement in disease progression through various mechanisms, such as increased cholesterol synthesis, reprogramming of lipid metabolism, sensitivity to PARP inhibitors, and modulation of the immune response [21,22].

Therapeutic strategies that co-target cholesterol homeostasis and PARP activity may exhibit synergistic effect against TNBC. PARP inhibitors not only induce BRCA-deficient tumor cell death but also potentiate antitumor immunity through multifaceted mechanisms. PARP inhibitors suppress antitumor immunity by upregulating PD-L1 expression, whereas PD-L1 blockade restores T cell-mediated cytotoxicity against PARPi-treated cancer cells, thereby overcoming therapy-induced immune evasion [23]. In addition, PARP inhibitors have been shown to promote the infiltration of CD8^+^ CAR-T cells in the TME by activating the cGAS/STING signaling pathway. This results in a shift in immunostimulatory signaling towards a pro-inflammatory state, which enables low-dose CAR-T cell treatment to induce effective tumor regression [24]. The PARP inhibitor Olaparib has been shown to enhance CD8^+^ T cell infiltration and activation in vivo. This effect is mediated via tumour cell cGAS/STING pathway activation and subsequent dendritic cell (DC) paracrine signaling, exhibiting greater potency in HR-deficient TNBC models [25].

In our previous research, we found that PARP inhibitors could induce ferroptosis in TNBC cells [26]. This study investigates how the PARP inhibitor reprograms lipid metabolism in TNBC cells, with a central focus on the cholesterol transport protein NPC1L1. We explore the potential benefits of co-targeting NPC1L1 and PARP inhibitors to enhance treatment efficacy by disrupting cholesterol homeostasis and amplifying lipid peroxidation. Furthermore, our findings demonstrate that combining PARP inhibitors with lipid-lowering agents (e.g., Ezetimibe) or AKT inhibitors (e.g., AZD5363) synergistically enhances ferroptosis induction through GSH depletion and MDA accumulation. Critically, we investigate how NPC1L1 inhibitors augment PARP inhibitor-driven immunogenic cell death in vivo, promoting CD8^+^ T cell infiltration and antitumor immunity. These insights may lead to innovative combination therapies that leverage lipid metabolism, aiming to surmount treatment resistance and enhance outcomes for TNBC patients.

## 2. Materials and Methods

### 2.1. Reagents

The following pharmacological inhibitors were utilized in this study: PARP inhibitors Olaparib (PubChem CID: 23725625; GlpBio, GC17580; Montclair, CA, USA), Niraparib (PubChem CID: 24958200; GlpBio, GC17802), Rucaparib (PubChem CID: 9931954; GlpBio, GC15955), and Talazoparib tosylate (PubChem CID: 135565654; GlpBio, GC37728); NPC1L1 inhibitor Ezetimibe (PubChem CID: 150311; GlpBio, GC15605); and AKT kinase inhibitor Capivasertib (AZD5363; PubChem CID: 25227436; GlpBio, GC11752).

The following antibodies were used: NPC1L1 (rabbit monoclonal, 1:1000; Abcam, Cat# ab284598, Lot No: GR342448-1; Cambridge, UK), RELA (rabbit monoclonal, 1:1000; Cell Signaling Technology, Cat# 8242, Lot No: 14; Danvers, MA, USA), GAPDH (mouse monoclonal, 1:5000; Proteintech, Cat# 60004-1-Ig, Lot No: 00066073; Rosemont, IL, USA), Anti-PARP (46D11) Rabbit mAb (1:1000; Cell Signaling Technology, Cat# 9532, Lot No: 10; Danvers, MA, USA), HRP-conjugated anti-β-actin (1:1000; Abcam, Cat# ab49900, Lot No: GR3457139-1; Cambridge, UK), Anti-PI3 Kinase p85 alpha (1:1000; Abcam, Cat# ab191606, Lot No: 1011480-6), Anti-mTOR (1:1000; Abcam, Cat# ab134903, Lot No: 1000631-7), Anti-AKT1 + AKT2 + AKT3 (1:1000; Abcam, Cat# ab179463, Lot No: 1010423-9), Anti-(phospho S472 + S473 + S474) AKT1 + AKT2 + AKT3 (1:1000; Abcam, Cat# ab192623, Lot No: 10081813-8), Anti-mouse IgG HRP-linked (1:5000; Cell Signaling Technology, Cat# 7076, Lot No: 36), and Anti-rabbit IgG HRP-linked (1:5000; Cell Signaling Technology, Cat# 7074, Lot No: 31).

### 2.2. Cell Culture

The human breast cancer cell lines MDA-MB-231 (triple-negative breast cancer, TNBC; ER^−^/PR^−^/HER2^−^; BRCA1 wild-type), MDA-MB-453 (HER2-enriched; ER^−^/PR^−^/HER2^+^; BRCA1 wild-type), and MDA-MB-468 (TNBC, basal-like; ER^−^/PR^−^/HER2^−^; BRCA1 wild-type) were obtained from the Cell Bank of Shanghai Institute of Biochemistry and Cell Biology (SIBCB, Shanghai, China). The BRCA1-mutated TNBC cell line HCC1937 (ER^−^/PR^−^/HER2^−^; BRCA1 5382insC) was purchased from Guangzhou Cellcook Biotechnology (Guangzhou, China). MDA-MB-231, MDA-MB-468, and HCC1937 cells were maintained in RPMI-1640 medium (Sigma-Aldrich, St. Louis, MO, USA, R8758) supplemented with 10% fetal bovine serum (FBS; Gibco™, Grand Island, NY, USA, 10099141) and 1% penicillin-streptomycin (HyClone™, Logan, UT, USA, SV30010), while MDA-MB-453 cells were cultured in DMEM/high glucose medium (HyClone™, Logan, UT, USA, SH30022.01) with 10% FBS. All cell lines were authenticated by short tandem repeat (STR) profiling and incubated at 37 °C in a humidified 5% CO_2_ atmosphere. BRCA1 status was verified through the COSMIC database.

### 2.3. Absolute Quantitative Lipidomics

Following 48-h treatment of MDA-MB-231 cells with Niraparib (25 μM), cells were cryopreserved in liquid nitrogen. Lipid extraction was initiated by spiking samples with 20 μL of internal lipid standards, followed by homogenization in 200 μL deuterium oxide and 240 μL methanol. After adding 800 μL methyl tert-butyl ether (MTBE), the mixture underwent ultrasound-assisted extraction for 20 min at 4 °C, equilibration (30 min, room temperature), and centrifugation (14,000× *g*, 15 min, 10 °C). The collected upper organic phase was dried under nitrogen and analyzed via LC-MS/MS using a C18 reversed-phase column with a gradient of solvent A (acetonitrile-water, 6:4, 0.1% formic acid, 0.1 mM ammonium formate) and solvent B (acetonitrile-isopropanol, 1:9, 0.1% formic acid, 0.1 mM ammonium formate) at 0.3 mL/min (30% B initial, 2 min; 100% B over 23 min; 5% B re-equilibration, 10 min). Lipids were identified/quantified using LipidSearch™ software (Thermo Scientific™, Waltham, MA, USA, v5.0) with reference to internal standards.

### 2.4. PCR Array and Quantitative Real-Time PCR Analysis

Total RNA was isolated using the FastPure^®^ Cell/Tissue Total RNA Isolation Kit (Vazyme, Nanjing, Jiangsu, China, RC112-01), and 1 μg RNA was reverse-transcribed to cDNA with HiScript^®^ II Q RT SuperMix (Vazyme, Nanjing, Jiangsu, China, R223-01). Fresh tumor tissues (50–100 mg) were homogenized in 1 mL TRIzol^®^ Reagent (Thermo Fisher Scientific, Waltham, MA, USA, Cat#15596026CN) using a Precellys^®^ 24 homogenizer (Bertin Instruments, Montigny-le-Bretonneux, France) at 5000 rpm for 30 s × 3 cycles. RNA extraction was performed following the TRIzol-chloroform protocol, which included phase separation with 0.2 mL chloroform, RNA precipitation using 0.5 mL isopropanol, and washing with 75% ethanol. Finally, RNA pellets were dissolved in 30 μL RNase-free water. The primer sequences used in the PCR experiments are shown in Table 1. For pathway screening, cDNA was mixed with ChamQ Universal SYBR qPCR Master Mix (Vazyme, Nanjing, Jiangsu, China, Q711-02) and loaded onto RT^2^ Profiler PCR Array plates (Qiagen, PAHS-343ZA, Hilden, Germany). Amplification was performed on a LightCycler^®^ 96 system (Roche, 04729692001, Mannheim, Germany) under standardized conditions: 1 cycle of pre-denaturation at 95 °C for 30 s, 40 cycles of denaturation at 95 °C for 10 s, and annealing/extension at 60 °C for 30 s, followed by a melting curve analysis (95 °C for 15 s, 60 °C for 60 s, and 95 °C for 15 s). Target validation used custom primers (Sangon Biotech, Shanghai, China) with identical SYBR reagents and cycling parameters. Data were normalized to GAPDH and analyzed via the 2^−ΔΔCt^ method.

### 2.5. Western Blot

Total proteins were extracted from harvested cell samples using RIPA lysis buffer, and protein concentrations were determined with a Pierce™ BCA Protein Assay Kit (Thermo Fisher Scientific, Waltham, MA, USA; #23225). Equal amounts of protein samples (20–30 μg) were loaded onto 10% SDS-PAGE gels for electrophoretic separation. Subsequently, proteins were transferred onto polyvinylidene fluoride (PVDF) membranes using a semi-dry transfer system. The membranes were blocked with 5% (*w*/*v*) skim milk in Tris-buffered saline containing 0.1% Tween 20 (TBST) for 1 h at room temperature, followed by overnight incubation with primary antibodies (diluted in TBST with 5% bovine serum albumin, BSA) at 4 °C. After three washes with TBST (10 min each), the membranes were incubated with horseradish peroxidase (HRP)-conjugated secondary antibodies (1:5000 dilution in TBST containing 5% skim milk) for 1 h at room temperature. Unbound secondary antibodies were removed by three additional TBST washes. Protein bands were visualized using enhanced chemiluminescence (ECL) substrate, and densitometric analysis was performed with ImageJ software (version 1.54p).

### 2.6. Dual-Luciferase Reporter Assay

Cells in the logarithmic growth phase were seeded into 24-well plates at 5 × 10^4^ cells/well in DMEM medium supplemented with 10% FBS. Each well was transfected with 500 ng of plasmid DNA (pGL4.32-NPC1L1-promoter firefly luciferase reporter or control vector), 1.5 μL Lipofectamine 3000 (Invitrogen, Carlsbad, CA, USA, L3000015), and 1 μL P3000 enhancer reagent in 50 μL Opti-MEM (Gibco™, Grand Island, NY, USA, 31985070). The mixture was incubated at room temperature for 15 min, followed by addition of 450 μL fresh medium. After 6 h of incubation, the transfection medium was replaced with 1 mL complete medium containing 10% FBS. Cells were lysed with 200 μL 1× Passive Lysis Buffer (Promega, Madison, WI, USA, E1941) for 15 min at room temperature with gentle agitation. Lysates were centrifuged (12,000× *g*, 2 min, RT), and 20 μL supernatant was transferred to a 96-well plate. A quantity of 100 μL Luciferase Assay Reagent II (Promega, Madison, WI, USA, E1910; containing 0.47 mM luciferin, 20 mM Tricine buffer) was added to measure firefly luciferase activity, followed by 100 μL Stop & Glo^®^ Reagent (Promega, Madison, WI, USA, E1910; 23.6 μM coelenterazine) for Renilla luciferase quantification using a GloMax^®^ Navigator luminometer (Promega, Madison, WI, USA). Firefly luciferase signals were normalized to Renilla values for data analysis.

### 2.7. Transcriptome Analysis

Total RNA was extracted from harvested cells using TRIzol^®^ Reagent (Invitrogen, Carlsbad, CA, USA, 15596026), followed by poly(A) mRNA enrichment via the NEBNext^®^ Poly(A) mRNA Magnetic Isolation Module (New England Biolabs, Ipswich, MA, USA, E7490). The purified mRNA was reverse-transcribed into cDNA using SuperScript™ IV Reverse Transcriptase (Invitrogen, Carlsbad, CA, USA, 18090010) with oligo(dT)20 primers. For library preparation, cDNA fragments were enzymatically fragmented and ligated to Illumina TruSeq^®^ adapters (Illumina, San Diego, CA, USA, 20015965) using the NEBNext^®^ Ultra™ II DNA Library Prep Kit (New England Biolabs, Ipswich, MA, USA, E7645), incorporating dual-index barcodes through 8 cycles of PCR amplification. Libraries were quantified via Qubit™ Fluorometry (Thermo Fisher Scientific, Waltham, MA, USA) and sequenced on an Illumina NovaSeq 6000 platform (150 bp paired-end reads, ~40 million reads per sample). Raw sequencing data were processed through FastQC for quality control, aligned to the human reference genome (GRCh38) using STAR (v2.7.9a), and quantified via featureCounts (v2.0.3). Differentially expressed genes (DEGs) were identified using DESeq2 (*p*adj < 0.05, |log2FC| > 1), followed by functional enrichment analysis via DAVID (v2023q1) and pathway mapping with KEGG [https://www.kegg.jp; (accessed on 22 August 2022)].

### 2.8. Total Cholesterol Measurement

Cellular cholesterol and cholesteryl ester levels were quantified using a Cholesterol/Cholesteryl Ester Assay Kit (Abcam, Cambridge, UK; Cat# ab65359), which specifically detects free cholesterol and total cholesterol (free + esterified) through enzymatic conversion. Method validation was performed by thin-layer chromatography (TLC) on a silica gel plate using a solvent system of hexane: diethyl ether: acetic acid (70:30:1, *v*/*v*/*v*) to confirm lipid extraction specificity.

Briefly, drug-treated cells were washed twice with ice-cold PBS, and lipids were extracted with 200 μL of chloroform: isopropanol:NP-40 (7:11:0.1, *v*/*v*/*v*) for 30 min at 4 °C. After centrifugation at 15,000× *g* for 10 min, the organic phase was collected and dried under nitrogen gas at 50 °C. Samples were further vacuum-dried for 30 min and reconstituted in 200 μL assay detection buffer. Cholesterol standards (0–100 μg/mL, provided with the kit) were processed in parallel to generate a calibration curve. The reaction mixture (samples or standards with cholesterol oxidase and peroxidase) was incubated at 37 °C for 60 min in the dark. Absorbance at 570 nm was measured using a microplate reader, and total cholesterol content was normalized to cellular protein concentration determined by BCA assay.

### 2.9. ATP Levels Determination

ATP levels in cell samples were quantified using an ATP assay kit (Beyotime Biotechnology, Shanghai, China; Cat# S0026). Briefly, 1 × 10^6^ cells per sample were collected by centrifugation (300× *g*, 5 min), lysed in 100 μL of ice-cold lysis buffer provided with the kit, and centrifuged at 12,000× *g* for 5 min at 4 °C to remove debris. A 20 μL aliquot of the supernatant was mixed with 100 μL of ATP detection working solution in a 96-well plate and incubated at room temperature for 10 min in the dark. ATP standards (0–10 μM, provided with the kit) were processed in parallel to generate a calibration curve. Luminescence was measured using a microplate reader, and ATP concentrations were normalized to total protein content quantified by BCA assay.

### 2.10. GSH Measurement and MDA Levels Analysis

Cells were cultured in 6-well plates under standard conditions (37 °C, 5% CO_2_) and harvested by trypsinization after treatment. Cellular glutathione (GSH) and oxidized glutathione (GSSG) levels were quantified using GSH and GSSG Assay Kit (Beyotime Biotechnology, Shanghai, China; Cat# S0053) following the manufacturer’s protocol. Briefly, cells were lysed in ice-cold assay buffer, centrifuged at 12,000× *g* for 10 min at 4 °C, and supernatants were incubated with 5,5′-dithiobis (2-nitrobenzoic acid) (DTNB) to measure total GSH. For GSSG analysis, reduced GSH was masked by pretreatment with 1-methyl-2-vinylpyridinium triflate (M2VP). Malondialdehyde (MDA) levels, reflecting lipid peroxidation, were determined using a Lipid Peroxidation MDA Assay Kit (Beyotime Biotechnology, Shanghai, China; Cat# S0131S). Cell lysates were reacted with thiobarbituric acid (TBA) at 95 °C for 40 min, and absorbance was measured at 532 nm using a microplate reader. Data were normalized to total protein concentration determined by BCA assay.

### 2.11. Flow Cytometry

Following drug treatment, cells were incubated with 2 μM BODIPY^®^ 581/591 C11 (Invitrogen, Carlsbad, CA, USA, D3861), a lipid peroxidation-sensitive fluorescent probe, in serum-free medium at 37 °C for 20 min in the dark. After incubation, cells were washed twice with ice-cold phosphate-buffered saline (PBS) and detached using 0.25% trypsin-EDTA (Gibco™, Grand Island, NY, USA). Cell suspensions were resuspended in PBS containing 2% FBS and analyzed immediately on a BD FACSCanto II flow cytometer (BD Biosciences, Franklin Lakes, NJ, USA) equipped with a 488 nm laser. Fluorescence emission was captured at 530 nm (oxidized BODIPY^®^ C11, green channel) and 590 nm (non-oxidized BODIPY^®^ C11, red channel). Relative lipid peroxidation levels were quantified as the ratio of green-to-red fluorescence intensity using FlowJo software (v10.8.1, BD Biosciences, Franklin Lakes, NJ, USA).

### 2.12. Cell Viability Assay

Cells were seeded in 96-well plates at 5 × 10^3^ cells/well in 200 μL of complete medium and incubated overnight at 37 °C under 5% CO_2_. After being treated with the inhibitors for 24 h, 0.5 mg/mL 3-(4,5-dimethylthiazol-2-yl)-2,5-di-phenyltetrazolium bromide (MTT, Sigma, DH343-1, St. Louis, MO, USA) was added to each well and incubated for 4 h. Then, the liquid in each well was removed, and 100 µL of DMSO was added. The absorbance was measured at 570 nm with a microplate reader (Thermo Scientific Multiskan FC, Waltham, MA, USA).

### 2.13. Gene Silencing Experiments

siRNA Transfection: Gene-targeting siRNAs and a scrambled control siRNA (siScramble) were chemically synthesized by Shanghai GenePharma Co., Ltd. (Shanghai, China). The siRNA sequences involved are shown in Table 2. TNBC cells were transfected with 20 nM siRNA using Lipofectamine^®^ 2000 (Invitrogen, 11668019) for 10 h, followed by replacement with complete medium. Cells were harvested 48 h post-transfection for downstream analysis.

Lentiviral shRNA Transduction: For stable knockdown, TNBC cells were transduced with lentiviral particles encoding RELA-specific shRNAs (MOI = 10) in RPMI-1640 medium containing 8 μg/mL polybrene (Sigma-Aldrich, St. Louis, MO, USA, TR-1003) for 48 h.The shRNA sequences involved are shown in Table 3. Stable clones were selected with 0.5–2 μg/mL puromycin (InvivoGen, San Diego, CA, USA, ant-pr-1) for 18 days. Knockdown efficiency was validated by Western blot.

### 2.14. T Cell Cytotoxic Assay

Peripheral blood mononuclear cells (PBMCs) were isolated from human subjects using Ficoll-Paque™ density gradient centrifugation (Cytiva, Uppsala, Sweden), counted with a hemocytometer, and seeded at 1 × 10^6^ cells/well in 24-well plates. Cells were activated with RPMI 1640 medium supplemented with 10 μg/mL anti-human CD3 antibody (BioLegend, San Diego, CA, USA; Cat# 300401), 10% fetal bovine serum (FBS), and 100 U/mL recombinant human IL-2 (PeproTech, Cranbury, NJ, USA; Cat# 200-02) for 3 days at 37 °C in 5% CO_2_. Activated T cells were collected by centrifugation (300× *g*, 10 min) and expanded. For effector-to-target (E:T) ratio optimization, tumor cells (e.g., MDA-MB-231) were labeled with 5 μM carboxyfluorescein succinimidyl ester (CFSE; Thermo Fisher Scientific, Waltham, MA, USA; Cat# C34554) by incubating 1 × 10^6^ cells in 1 mL PBS containing CFSE at 37 °C for 15 min, followed by two washes with complete medium. Labeled tumor cells (5 × 10^4^ cells/well) were seeded in 12-well plates, pretreated with drugs or vehicle for 72 h, and co-cultured with activated PBMCs for 24 h. Cells were detached using 0.25% trypsin-EDTA (Gibco™, Grand Island, NY, USA), washed with PBS, resuspended in flow cytometry buffer (PBS with 2% FBS), and analyzed on a BD FACSCanto II flow cytometer (BD Biosciences, Franklin Lakes, NJ, USA). Tumor cell apoptosis was quantified by gating CFSE-positive populations and staining with Annexin V-PE/7-AAD (BD Pharmingen™, San Diego, CA, USA; Cat# 559763).

### 2.15. Syngeneic 4T1 Mammary Tumor Model

All animal experiments were approved by the Laboratory Animal Welfare & Ethics Committee of Fujian Medical University (approval No. FJMU IACUC 2020–0076). Female BALB/c mice (5–6 weeks) were purchased from Vital River Laboratories (Beijing, China; license no. SCXK2019–0008) and housed in specific pathogen-free SPF cages under standard condition (temperature: 19–23 °C, 12 h light/dark cycle, and 5 mice per cage). For tumor induction, 1 × 10^6^ 4T1 cells in 100 μL PBS (cell viability >90% by trypan blue exclusion) were subcutaneously inoculated into the dorsal flank of each mouse (*n* = 49). Tumor volume was monitored using digital calipers and calculated as volume = length × (width/2)^2^. When tumors reached 100 mm^3^, mice were randomized into 7 groups (*n* = 7/group) and treated daily with Niraparib (25 mg/kg), AZD5363 (8 mg/kg), or Ezetimibe (50 mg/kg) dissolved in a vehicle containing 5% DMSO, 40% PEG300, 5% Tween 80, and 50% normal saline. All mice were euthanized via intraperitoneal injection of 2% pentobarbital sodium (100 mg/kg) after 18 days.

### 2.16. Paraffin Section Immunohistochemistry Experiment

Tumor tissue samples were fixed in 4% neutral-buffered formalin at room temperature for 24–48 h, embedded in paraffin, and sectioned into 4–5 μm slices, which were mounted onto glass slides. To address antigen masking, heat-induced epitope retrieval was performed by immersing the slides in 10 mM sodium citrate buffer (pH 6.0) and heating them in a pressure cooker at 95–100 °C for 20 min, followed by gradual cooling to room temperature. Non-specific binding was blocked by incubating the sections with a PBS solution containing 5% normal goat serum and 0.1% Triton X-100 for 1 h at 25 °C. Primary antibodies—CD8 and Granzyme B—were diluted in Dako antibody diluent and applied to the sections, which were incubated overnight at 4 °C in a humidified chamber. After washing three times with PBST (0.05% Tween-20), the slides were incubated with HRP-conjugated goat anti-mouse IgG (H + L) secondary antibody (Thermo Fisher Scientific #31430, 1:500 dilution) for 1 h at 25 °C. Staining was developed using DAB substrate for 5–10 min, followed by counterstaining with Mayer’s hematoxylin for 1 min. The sections were dehydrated through a graded ethanol series (70%, 95%, and 100% ethanol; 2 min each), cleared in xylene, and mounted with Permount™ mounting medium. Images were acquired and analyzed using a Nikon Eclipse E800 microscope (Nikon Corporation, Tokyo, Japan) equipped with NIS-Elements imaging software (version 5.21.01).

### 2.17. Statistical Analysis

Data were analyzed using SPSS 19.0. Continuous variables were expressed as mean ± standard deviation (SD). Independent two-sample *t*-test (Student’s *t*-test, *p* < 0.05) was used for comparisons between two groups. For multi-group comparisons, homogeneity of variance was first assessed via Levene’s test. If variances were homogeneous, one-way ANOVA followed by Tukey’s HSD (honestly significant difference) or LSD (least significant difference) post hoc tests was applied for pairwise comparisons. For inhomogeneous variances, nonparametric Kruskal–Wallis H test was employed.

## 3. Results

### 3.1. Lipid Metabolism Significantly Altered by PARP Inhibitors

**Figure 1:**
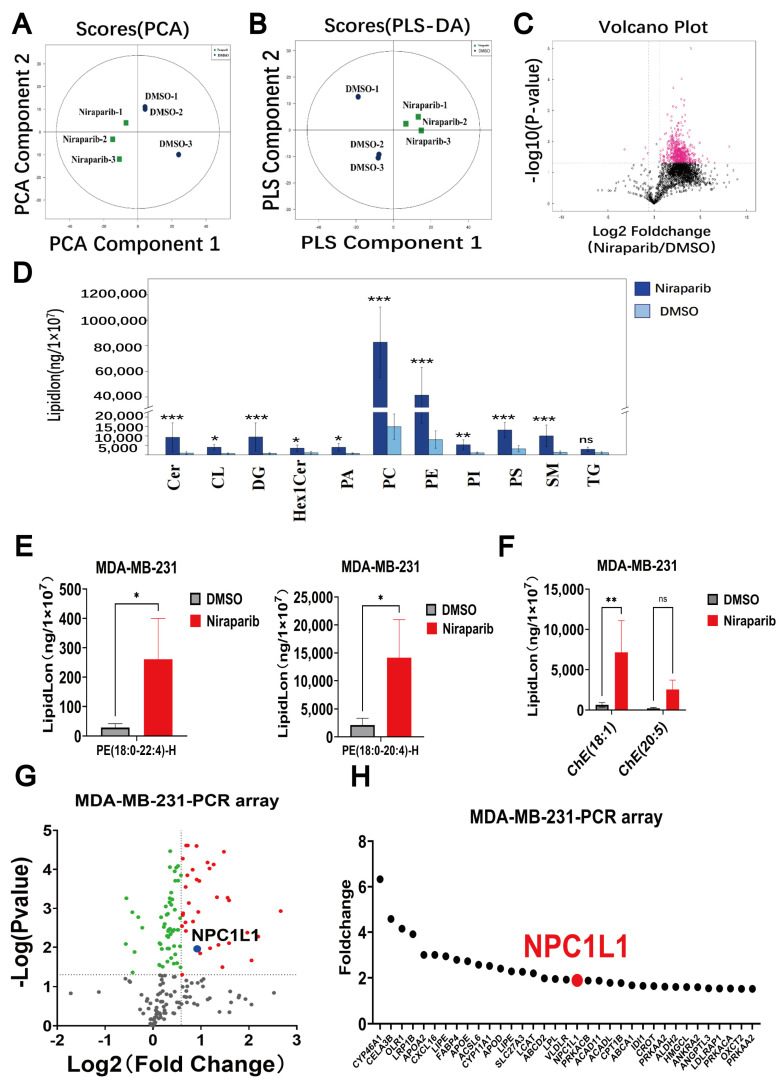
The abnormal lipid accumulation induced by Niraparib treatment in cancer cells. The human TNBC cell line MDA-MB-231 was treated with 25 μM Niraparib for 48 h, and the metabolites were extracted for non-targeted lipidomic detection. (**A**) Unsupervised PCA of the Niraparib-treated groups and the negative control groups. The model parameters are R^2^X = 0.719, green: Niraparib-1, blue: DMSO. (**B**) PLS-DA analysis of these groups. The model parameters are R^2^X = 0.792, R^2^Y = 0.999, and Q^2^ = 0.943, green: Niraparib-1, blue: DMSO. (**C**) Volcano plot of abundant lipid metabolites in MDA-MB-231 cells between the Niraparib-treated and control groups. Significantly up-regulated metabolites are shown in red. (**D**) The changes in the relative amounts of the most important lipid classes in the cells were shown after treatment with Niraparib. Lipid classes on the *X*-axis: Cer (Ceramide); CL (Cardiolipin); DG (Diacylglycerol); HexCer (Hexosylceramide); PA (Phosphatidic acid); PC (Phosphatidylcholine); PE (Phosphatidylethanolamine); PI (Phosphatidylinositol); PS (Phosphatidylserine); SM (Sphingomyelin); TG (Triacylglycerol). *Y*-axis: LipidLon indicates the adduct ion forms of lipid molecules. (**E**) The levels of PE (18:0_20:4)-H and PE (18:0_22:4)-H were significantly upregulated. Error bars denote standard error from three individual experiments. (**F**) Lipidomics quantification of ChE (18:1) and ChE (20:5). ChE is the abbreviation for cholesterol ester. Error bars denote standard error from three individual experiments. (**G**) The volcano plot of PCR array data shows significant changes in genes in MDA-MB-231 breast cancer cells after treatment with PARP inhibitor Niraparib. In the volcano plot, red dots represent genes with a fold change greater than 1.5 and a *p* value less than 0.05, with NPC1L1 highlighted in blue. Green dots represent genes with a fold change less than 1.5 and a *p* value less than 0.05, while gray dots represent genes with a *p* value less than 0.05. (**H**) GraphPad Prism software (version 10.4.2) displays PCR array data. Statistical significance between groups is indicated (* *p* < 0.05; ** *p* < 0.01; *** *p* < 0.001, ns: not significant).

To elucidate the mechanisms underlying PARP inhibitor-induced ferroptosis, we employed a cutting-edge lipidomics analysis platform based on mass spectrometry. We conducted a comprehensive lipid profiling analysis of samples treated with the PARP inhibitor Niraparib and compared them with control samples to determine the absolute quantities of various lipid molecules. Principal component analysis (PCA) was applied to each sample cohort to assess variability within and between groups. As shown in Figure 1A, data points of the same color represent different biological replicates, and their distribution illustrates the differences between the two groups within a 95% confidence interval. After sevenfold cross-validation, the R^2^X (cum) parameter of the PCA model reached 0.719, indicating the cumulative variance explained by the model. An R^2^X value closer to 1 suggests a more stable and reliable model.

Subsequently, we utilized partial least squares discriminant analysis (PLS-DA) to construct a model correlating lipid expression levels with sample classification. PLS-DA is a supervised discriminant analysis statistical method designed to predict sample categories. The PLS-DA score plot (Figure 1B) clearly delineates the differences between the Niraparib-treated group and the control group. Three key parameters highlight the model’s effectiveness and robustness: R^2^X (cum), which quantifies the explanatory power for the X variables; R^2^Y (cum), indicating the explanatory power for the Y variables; and Q^2^ (cum), representing the model’s predictive accuracy. The results were 0.792, 0.999, and 0.943, respectively. The model evaluation parameter Q^2^, obtained through sevenfold cross-validation, reached 0.943. Generally, a Q^2^ value greater than 0.5 indicates a stable and reliable model (Figure 1B). The PLS-DA model not only demonstrated commendable predictive ability and stability but also revealed that, compared to the control group, treatment with Niraparib significantly induced lipid metabolism reprogramming in the human TNBC cell line MDA-MB-231.

A comprehensive lipidomic analysis was conducted, employing fold change (FC) analysis as a univariate statistical method to discern differential lipid metabolites between the two sample cohorts. The differential analysis outcomes for all detected lipid species were depicted in volcano plots (Figure 1C), revealing a predominant localization of differential lipid molecules to the right, indicative of a substantial increase in lipid accumulation in TNBC cells following Niraparib treatment. Given the significance of lipid subtypes in defining biological functions, distinct from polar metabolites like amino acids and nucleotides, the expression variations of these subtypes were instrumental in identifying key lipid subtypes potentially implicated in the modulation of lipid metabolism by PARP inhibitors. The bar chart in Figure 1D visually delineated the content disparities among each lipid subtype, with the exclusion of low-abundance components. Notably, phosphatidylcholine (PC) and phosphatidylethanolamine (PE) emerged as both the most abundant and the most significantly upregulated by Niraparib. This is particularly relevant considering the fatty acid-rich microenvironment typically surrounding breast cancer cells due to the presence of adipocytes, which is known to influence breast cancer growth [27,28].

Researches have shown that PUFAs are susceptible to lipid peroxidation, and the potential for ferroptosis in cells is determined by the amount and location of free PUFAs [29]. In particular, PE containing arachidonic acid (C20:4) and its elongated metabolite adrenic acid (C22:4) are considered to be the major lipid peroxidation substrates leading to ferroptosis [30,31]. Consistent with the ferroptosis-inducing effect of PARP inhibitors, lipid quantification highlighted a significant upregulation of PE (18:0_20:4)-H and PE (18:0_22:4)-H species, which are implicated in driving cellular ferroptosis, with fold increases of 6.72 and 8.97, respectively, post Niraparib treatment (Figure 1E). Niraparib induced a notable upregulation of ChE (18:1) (Figure 1F), suggesting that PARP inhibitors could trigger abnormal cholesterol accumulation in TNBC cells.

Further investigation using a PCR array focused on lipoprotein signaling, cholesterol metabolism, and fatty acid metabolism pathways was performed to assess mRNA changes in lipid metabolism-related proteins in TNBC cells (MDA-MB-231) treated with Niraparib. The results were graphically represented in a volcano plot (Figure 1G), identifying 35 proteins with a statistically significant upregulation of over 1.5-fold (log2 fold change ≥ 0.58496, *p* value ≤ 0.05). Of particular interest was the cholesterol transport protein NPC1L1, which could mediate cholesterol uptake and was found to be significantly upregulated by 1.89-fold (*p* = 0.01) (Figure 1H). Despite the more pronounced upregulation of CYP46A1 in cholesterol turnover, we chose NPC1L1 as the core focus of our study based on the following multidimensional evidence chain. NPC1L1 is a crucial protein for intestinal cholesterol absorption and is closely associated with cancer, influencing the development of various types of cancer, including colorectal cancer, head and neck squamous cell carcinoma, ovarian cancer, and hepatocellular carcinoma [20]. It is worth noting that the genetic proxy inhibition of NPC1L1 is significantly associated with a reduced risk of breast cancer [21]. Further, the NPC1L1 inhibitor Ezetimibe was reported to significantly reduce the amount of ChE secreted at the basolateral membrane [32]. CYP46A1 catalyzes the conversion of brain cholesterol to 24S-hydroxycholesterol, and most studies have focused on Alzheimer’s disease. The mechanisms in solid tumors are unclear, and there are no cohort studies in cancer to support its clinical relevance. More importantly, there are no clinical-grade inhibitors targeting CYP46A1. Although CYP46A1 shows more significant changes at the mRNA level, its biological function, translational medicine value, and mechanistic operability are all inferior to those of NPC1L1. The selection of NPC1L1 as the core target in this study is based on a systematic evaluation of clinical evidence and the availability of tool compounds. Further researches are warranted to elucidate the regulatory mechanisms of NPC1L1 by PARP inhibitors in TNBC cells.

### 3.2. PARP Inhibitors Regulate the PARP1-RELA-NPC1L1 Signaling Axis

**Figure 2:**
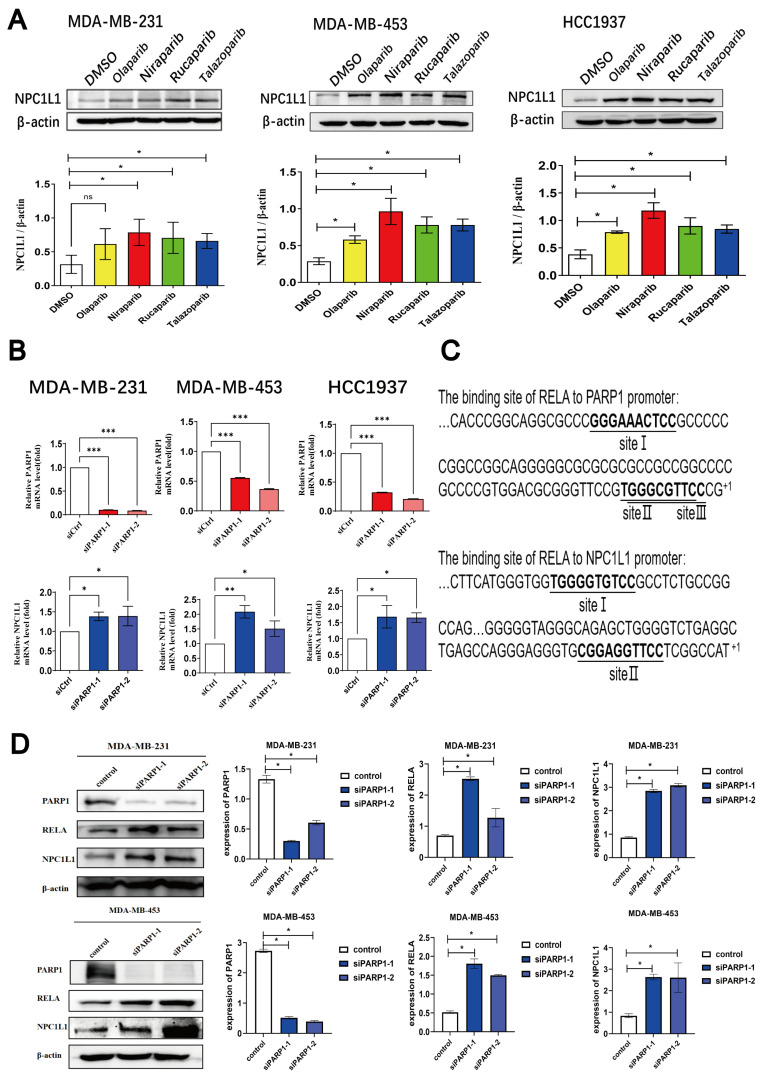

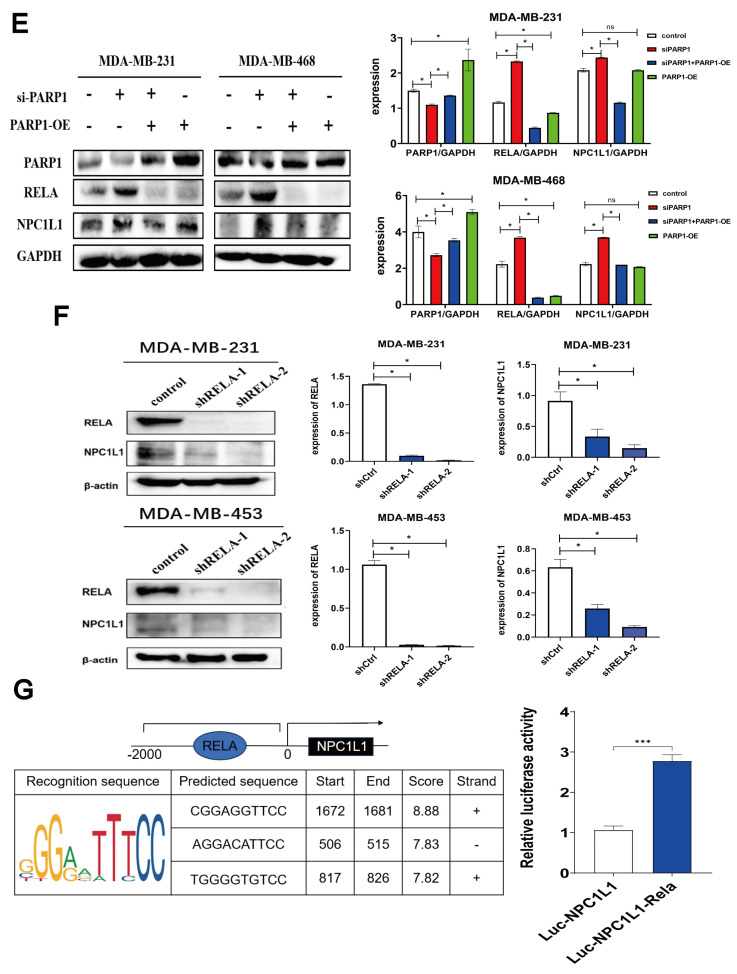
PARP inhibitors regulate the PARP1-RELA-NPC1L1 signaling axis. (**A**) Three TNBC cell lines were treated with four clinical PARPis (40 μM) for 48 h. The protein expression levels of NPC1L1 in these cells were analyzed by immunoblotting, with each blot containing three samples from the respective cell lines. (**B**) The mRNA levels of NPC1L1 were analyzed after transfection with siCtrl negative control or siPARP1 for 48 h in three TNBC cell lines. (**C**) JASPAR predicted the binding sites of the transcription factor RELA in the CDS region with the PARP1 promoter region, as well as the binding site of the CDS region of RELA with the NPC1L1 promoter region. (**D**) MDA-MB-231 and MDA-MB-453 cells were transfected with siCtrl negative control or siPARP, and 48 h later, the protein expression of NPC1L1, PARP1, and RELA was analyzed by immunoblotting. (**E**) Four experimental groups—control, si-PARP1, si-PARP1 + PARP1-OE, and PARP1-OE—were established to analyze the expression levels of PARP1, RELA, and NPC1L1 in cells. (**F**) MDA-MB-231 and MDA-MB-453 cells were transfected with siCtrl negative control or shRELA; the protein expression of NPC1L1, PARP1, and RELA were analyzed by immunoblotting. (**G**) The interaction between RELA and NPC1L1 was confirmed using a dual-luciferase reporter assay in 293T cells, providing a robust validation of the regulatory targeting. Statistical significance between groups is indicated (* *p* < 0.05; ** *p* < 0.01; *** *p* < 0.001, ns: not significant).

The PCR array data revealed that PARP inhibitors have the capacity to upregulate NPC1L1 mRNA levels. Subsequent studies were conducted to examine the influence of PARP inhibitors on NPC1L1 at the protein level. A panel of four clinically utilized PARP inhibitors was employed to treat three distinct TNBC cell lines, followed by an assessment of NPC1L1 protein levels. The findings indicated a consistent upregulation of NPC1L1 protein expression in response to all tested PARP inhibitors (Figure 2A). Furthermore, the transfection of TNBC cells with various siRNA sequences designed to knock down PARP1 also resulted in elevated NPC1L1 mRNA levels (Figure 2B). These observations suggest that both pharmacological inhibition and genetic suppression of PARP1 in TNBC cells contribute to the upregulation of NPC1L1.

To delve into the molecular underpinnings of how PARP inhibitors enhance NPC1L1 expression, we utilized the bioinformatics tool JASPAR to ascertain the presence of any binding sites between PARP1 and the NPC1L1 promoter region. The findings from this analysis suggested an absence of direct binding sites, leading us to hypothesize that PARP1 modulates NPC1L1 expression indirectly, potentially via the intermediation of other transcription factors. Some studies have indicated that SIRT1, an NAD^+^-dependent protein deacetylase, can interact with the NF-κB RELA/p65 subunit, leading to the deacetylation of p65 at Lys310, which in turn suppresses the transcriptional activity of NF-κB. PARP-1 inhibits the activity of SIRT1 by competing with it for the shared substrate NAD^+^, contributing to an inflammatory response [33]. Based on this, we propose the hypothesis that PARP1 may modulate the expression of NPC1L1 via RELA. Utilizing JASPAR for prediction, we identified 3 potential binding sites between RELA and the PARP1 promoter region, as well as 2 binding sites between RELA and the NPC1L1 promoter region (Figure 2C). Subsequent to knocking down PARP1 in two TNBC cell lines with siRNA sequences of different specificities, we observed that the reduction in PARP1 expression corresponded with an increase in both NPC1L1 and RELA protein levels (Figure 2D). To delve deeper into the regulatory mechanism of PARP1 on RELA and NCP1L1, we established four groups: control group, si-PARP1 group, si-PARP1 + PARP1-OE group, and PARP1-OE group. We detected the expression of PARP1, RELA, and NCP1L1 in the cells and found that after knocking down PARP1, the proteins RELA and NCP1L1 were upregulated. Upon the addition of PARP1-OE, the proteins RELA and NCP1L1 were downregulated, which fully demonstrated the regulatory mechanism by which knocking down PARP1 upregulates RELA and NCP1L1(Figure 2E).

To ascertain the regulatory role of RELA on NPC1L1, shRNA plasmids with distinct sequences were transfected into two TNBC cell lines to knockdown RELA. Our findings indicated a concordant decrease in NPC1L1 protein levels alongside the suppression of RELA (Figure 2F). JASPAR analysis predicted two specific binding sites for the transcription factor RELA within the NPC1L1 promoter region. Consequently, a 2-kilobase sequence upstream of the NPC1L1 mRNA transcription start site in the 5′-flanking region was cloned into the PGL3-Basic vector, positioning it upstream of the luciferase reporter gene. This construct was co-transfected with an overexpression plasmid harboring the RELA gene’s coding sequence (CDS). A dual-luciferase reporter assay was then employed to assess the direct regulatory effect of RELA on NPC1L1 mRNA expression. The results showed that NPC1L1 is transcriptionally up-regulated by RELA (Figure 2G). Based on the aforementioned results, it can be concluded that the inhibition of PARP1 leads to the up-regulation of RELA, which in turn further promotes the expression of NPC1L1.

### 3.3. PARP Inhibitors Activate the AKT Signaling Pathway Through NPC1L1

**Figure 3:**
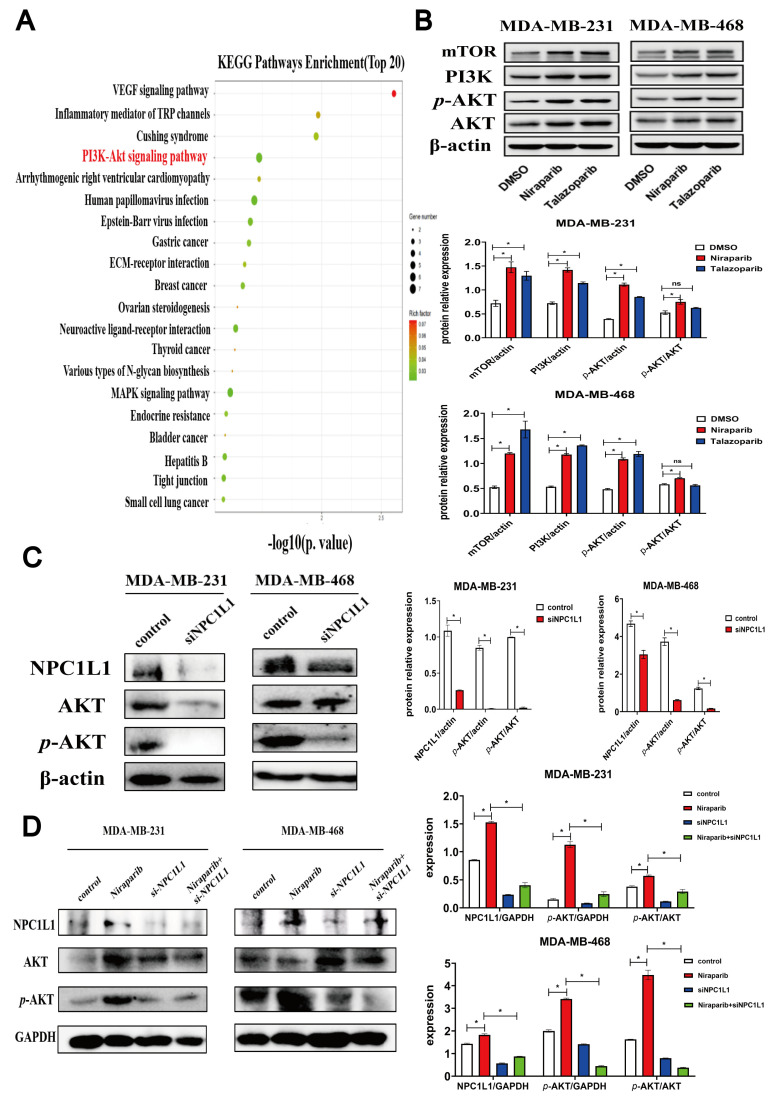
PARP inhibitors activate the AKT signaling pathway through NPC1L1. (**A**) KEGG enrichment analysis in transcriptomic data. The significant expression changes of 20 pathways were performed using the R package cluster Profiler (version 4.14.6). The PI3K-Akt signaling pathway, which is highlighted in red, is the focus of this study. (**B**) Representative expression levels of mTOR, PI3K, *p*-AKT, and AKT in MDA-MB-231 or MDA-MB-468 cells after incubation with DMSO, 25 μM Niraparib or Talazoparib for 48 h. β-actin was used as a loading control. (**C**) In the TNBC cell lines MDA-MB-231 and MDA-MB-468, NPC1L1 was knocked down using siRNA. The protein levels of NPC1L1, AKT, and *p*-AKT were then examined via Western blot analysis, with β-actin serving as an internal reference to normalize for protein loading. (**D**) NPC1L1 knockdown abrogates Niraparib-induced AKT pathway activation in TNBC cells, as demonstrated by comparative analysis of NPC1L1, AKT, and *p*-AKT expression across four experimental groups: control, Niraparib-treated, si-NPC1L1-transfected, and Niraparib + si-NPC1L1 combination. Statistical significance between groups is indicated (* *p* < 0.05, ns: not significant).

To delve deeper into the influence of NPC1L1 on signaling pathways in TNBC cells, we conducted a transcriptomic analysis. Our findings indicate that Niraparib notably enhances activation of the PI3K-AKT signaling pathway in TNBC cells, as illustrated in Figure 3A, where it is among the top 20 pathways in terms of signal enrichment. Given the frequent activation of the PI3K-AKT pathway in cancer and its significant upregulation by PARP inhibitors in TNBC, as shown in Figure 3B, the specific role of NPC1L1 in this activation process warrants further investigation. To explore this, we knocked down NPC1L1 in the TNBC cell lines MDA-MB-231 and MDA-MB-468 using siRNA. This intervention led to a marked reduction in the levels of phosphorylated AKT (*p*-AKT) protein, as depicted in Figure 3C. We observed that siNPC1L1 reduced AKT expression in MDA-MB-231 cells but not in MDA-MB-468 cells, which may reflect the biological heterogeneity between these cell lines. This discrepancy could be attributed to differences in AKT isoform expression and function, the complexity of the PI3K/AKT signaling pathway, and the distinct roles of NPC1L1 in cholesterol metabolism and transcriptional regulation in each cell line. AKT has multiple isoforms (such as AKT1, AKT2, and AKT3), which have different functions and expression levels in different cell lines. In some cell lines, when one AKT isoform is inhibited, other isoforms may maintain normal cellular function through compensatory mechanisms. For example, in MDA-MB-468 cells, even if siNPC1L1 inhibits a certain AKT isoform, other isoforms may still be able to maintain sufficient AKT activity, resulting in no significant decrease in total AKT expression levels. Given that changes in the phosphorylation status of AKT directly reflect the activation level of the signaling pathway, knocking down NPC1L1 in TNBC can inhibit the expression of *p*-AKT, thereby indicating that silencing NPC1L1 can suppress the AKT signaling pathway. To further explore whether knockdown of NPC1L1 can reverse the activation of the AKT signaling pathway induced by Niraparib, we set up four groups: control group, Niraparib group, si-NPC1L1 group, and Niraparib + si-NPC1L1 group. We detected the expression of NPC1L1, AKT, and *p*-AKT in the cells and found that knockdown of NPC1L1 can reverse the activation of the AKT signaling pathway induced by Niraparib (Figure 3D). These results suggested that while the PI3K-AKT signaling pathway was significantly activated by PARP inhibitors in TNBC, its activity could be substantially repressed by silencing NPC1L1. In summary, in TNBC cells, PARP inhibitors can suppress the PARP1-RELA-NPC1L1 signaling axis and regulate the AKT signaling pathway through NPC1L1.

### 3.4. NPC1L1 or AKT Inhibition Synergizes with PARP Inhibitors to Suppress TNBC Proliferation

**Figure 4:**
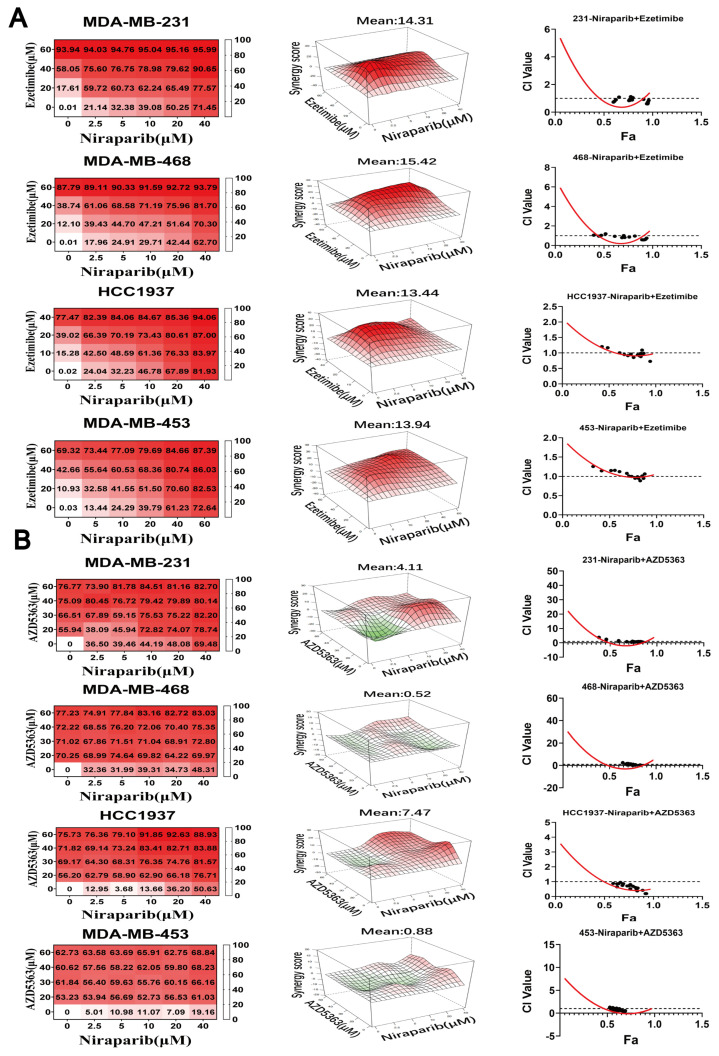
Combining NPC1L1 or AKT inhibition with PARP inhibitors can effectively suppress TNBC proliferation. (**A**,**B**) The different kinds of TNBC cells were treated with the drug combination for 72 h, and cell inhibition was assessed using the MTT assay. The degree of combination synergy is quantified by highest single agent (HSA) model, and the summary synergy score for a given combination was calculated by averaging all the measurements taken for the combination. Simulate the synergy index curve of the combined use of two drugs using CompuSyn software. Experiments were performed at least in quintuplicate.

NPC1L1 plays a crucial role in lipid metabolism reprogramming induced by PARP1 inhibitors. In our pursuit of combination strategies, we selected Ezetimibe, a specific NPC1L1 inhibitor, and employed a dose–response matrix to systematically evaluate the synergistic potential of drug combinations. Additionally, we utilized the highest single agent (HSA) model to assess the degree of drug synergy [34,35]. Figure 4A illustrates the synergistic effects of Niraparib and Ezetimibe across various TNBC cell lines, with dose–response data presented in matrix form. Synergistic dose regions are distinctly marked in red in the 3D co-plot. A synergy score exceeding 10 indicates a strong synergistic interaction between the two drugs. The combination inhibition rate of Niraparib and Ezetimibe demonstrates significant cytotoxicity against breast cancer cells, with a maximum inhibition rate reaching 95.99%. The IC_50_ values for monotherapy and the combination of the two drugs in TNBC cell lines are shown in Table 4. The data indicate that the IC_50_ values for TNBC cells were reduced when Niraparib and Ezetimibe were used in combination compared to monotherapy. The reduction in IC_50_ values upon combination treatment indicates the degree of synergy achieved. For example, in the MDA-MB-231 cell line, the IC_50_ of Niraparib was reduced from 20.52 μM to 9.18 μM when combined with Ezetimibe, representing a 55.2% reduction. Similarly, the IC_50_ of Ezetimibe was reduced from 31.76 μM to 13.77 μM, indicating a 56.6% reduction. These reductions highlight the substantial synergistic effect of the combination therapy. Furthermore, we employed the CompuSyn software (version 1.0.1) and the Chou–Talalay method to quantitatively assess drug synergy. A Combination Index (CI) curve was generated, where the *x*-axis (Fa) represents the fraction of proliferating cells inhibited, and the *y*-axis (CI) indicates the degree of synergy (CI < 1: synergy; CI = 1: additive effect; CI > 1: antagonism). These findings suggest that combining PARP inhibitors with lipid-modulating drugs, such as Ezetimibe, offers a promising new therapeutic strategy for treating TNBC.

Given that PARP inhibitors can activate the AKT signaling pathway via NPC1L1, we explored the potential sensitizing effect of combining the AKT inhibitor Capivasertib (AZD5363) with Niraparib on PARP inhibitors’ efficacy. AZD5363 is a pan-AKT kinase inhibitor with a proven track record in both monotherapy and combination therapy for breast cancer [36]. There have been reports of its combination with Olaparib in clinical studies [37], and its effectiveness in combination therapy for TNBC with PARP inhibitors is worth further investigation. Our combination drug experiments demonstrated promising therapeutic outcomes when AZD5363 and Niraparib were administered together in four TNBC cell lines. Notably, AZD5363 achieved higher cell inhibition rates at relatively lower concentrations. The IC_50_ values for monotherapy and the combination of the two drugs in TNBC cell lines are shown in Table 4. As indicated by the data, the combination of Niraparib and AZD5363 significantly reduced the IC_50_ values of Niraparib compared to monotherapy. Using the CompuSyn software, we simulated the CI curve for the drug combinations. The curves revealed that CI values decreased with increasing Fa, indicating enhanced synergy (Figure 4B). The extent of IC_50_ reduction upon combination treatment quantifies the degree of synergy. For instance, in the MDA-MB-231 cell line, the IC_50_ of Niraparib was reduced from 20.52 μM to 7.84 μM when combined with AZD5363, representing a 61.8% reduction. Similarly, in the MDA-MB-468 cell line, the IC_50_ of Niraparib was reduced from 23.79 μM to 2.04 μM, indicating a remarkable 91.4% reduction. These substantial reductions in IC_50_ values highlight the potent synergistic effects achieved with the combination of AZD5363 and Niraparib. Collectively, these data validate that AZD5363 can enhance the cytotoxicity of Niraparib in TNBC cells. Therefore, the combination of either Ezetimibe or AZD5363 with Niraparib for treating TNBC warrants further investigation.

### 3.5. Co-Targeting PARP and NPC1L1 or AKT Signaling Vulnerabilities Triggers Synergistic Ferroptosis in TNBC

**Figure 5:**
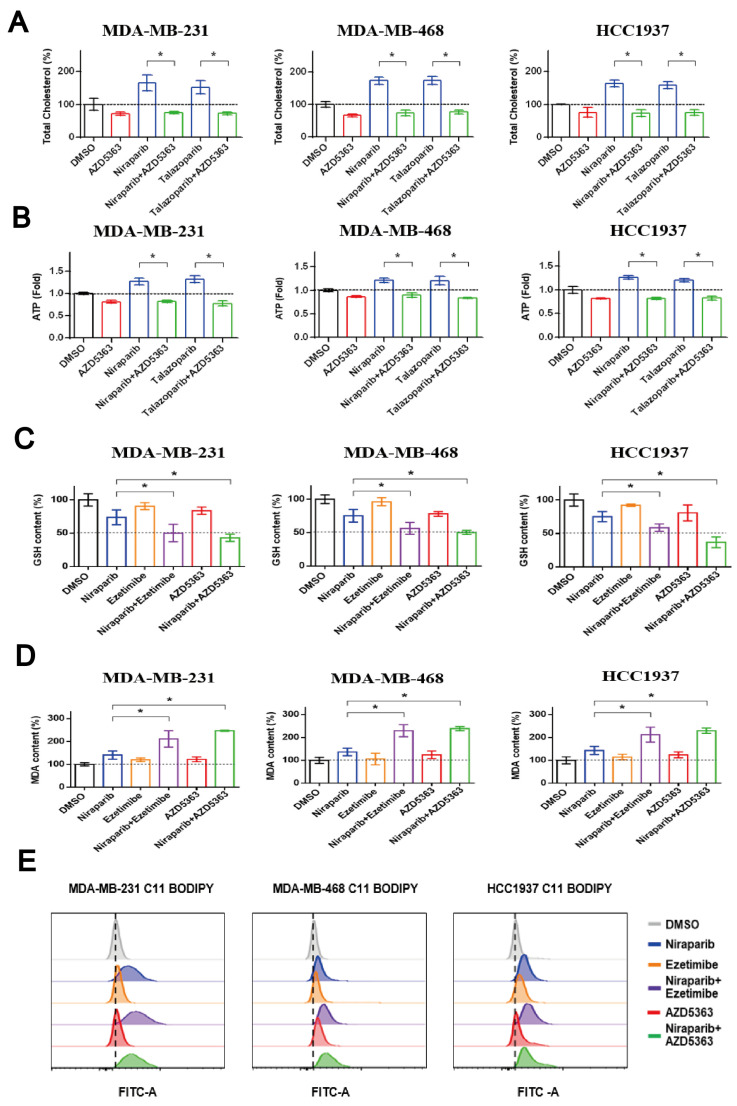
The combined treatment of PARP inhibitors and NPC1L1 inhibitors or AKT inhibitors can synergistically induce ferroptosis in TNBC cells. (**A**,**B**) MDA-MB-231 or HCC1937 cells were exposed for 48 h to PARP inhibitors (25 μM) and AKT inhibitor AZD5363 (5 μM). MDA-MB-468 cells were treated with the combination of 25 μM PARP inhibitors and 1 μM AZD5363 for 48 h. Total cellular cholesterol was measured by the cholesterol/cholesterol ester test kit, and ATP content was estimated using the ATP assay kit according to the instructions of each manufacturer. (**C**,**D**) Cells were exposed for 48 h to single agents or drug combination, and the ferroptosis biomarkers present in TNBC cells were detected using the colorimetric assay kits following the manufacturer’s instructions. MDA and GSH contents were determined by spectrophotometry. (**E**) The amount of lipid peroxides in cellular membranes was detected using BODIPY-C11 probe and flow cytometry. Statistical significance between groups is indicated (* *p* < 0.05).

We subsequently employed the AKT inhibitor AZD5363 to examine the impact of PARP inhibitors on cholesterol and ATP levels. Our findings indicated that AZD5363 could reverse the elevation in total intracellular cholesterol and ATP levels caused by Niraparib or Talazoparib (Figure 5A,B). This suggests that PARP inhibitors may facilitate cholesterol accumulation and ATP synthesis through the activation of the PI3K/AKT pathway. In this study, we investigated whether AZD5363 was similar to the combination of lipid-regulating drugs that makes breast cancer cells more sensitive to ferroptosis. The lipidomics revealed that Niraparib treatment increased the levels of MUFAs, which could inhibit ferroptosis by replacing PUFAs in cell membranes; the levels of the enzymes involved in MUFAs synthesis were first investigated. Moreover, the combination of PARP inhibitors with either AZD5363 or the cholesterol absorption inhibitor Ezetimibe significantly depleted glutathione levels and heightened lipid peroxidation in three TNBC cell lines (Figure 5C,D). Concurrently, the levels of lipid ROS, a marker of ferroptosis, were substantially elevated with these drug combinations (Figure 5E). The collective findings imply that PARP inhibitors might induce ferroptosis, a process that could be sensitized when combined with AKT inhibitors or lipid-regulating drugs.

### 3.6. The Combination of PARP Inhibitor and NPC1L1 Inhibitor Enhances the Killing Effect of T Cells on Tumor Cells

**Figure 6:**
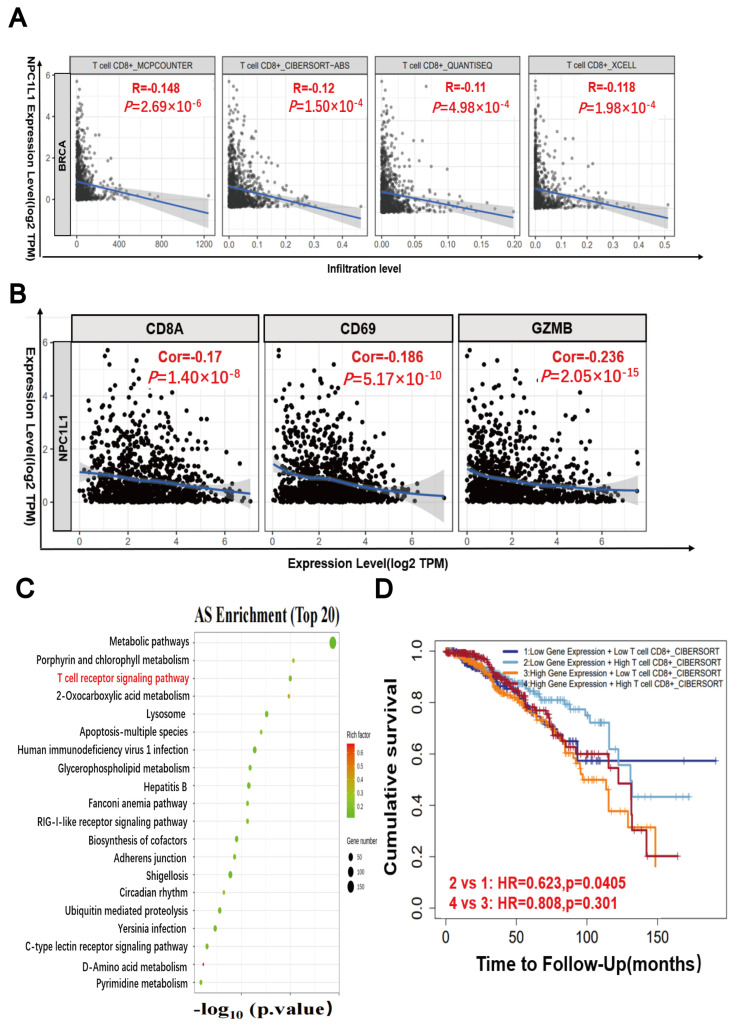

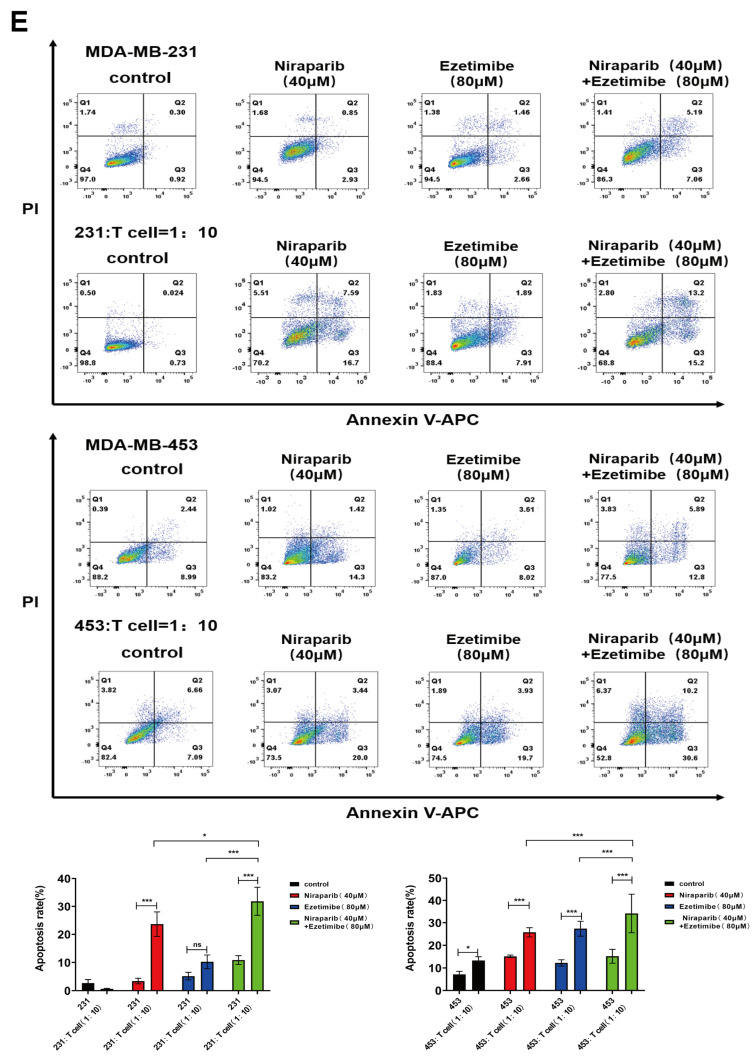
The T cell cytotoxicity induced by Niraparib can be enhanced by Ezetimibe. (**A**) The effect of NPC1L1 on CD8^+^ T cell infiltration in breast cancer cells was analyzed by TIMER database. (**B**) The correlation between NPC1L1 and CD8^+^ T cell activity indexes CD8A, CD69, GZMB in breast cancer cells was analyzed by TIMER database. (**C**) Transcriptome sequencing analysis of MDA-MB-231 cells treated with 25 μM Niraparib for 48 h. (**D**) The impact of NPC1L1 expression and CD8^+^ T cell infiltration on survival was analyzed by CIBERSORT algorithm in TIMER database. (**E**) MDA-MB-231 and MDA-MB-453 breast cancer cells were treated with Niraparib (40 μM), Ezetimibe (80 μM), or their combination for 24 h. After treatment, CFSE-labeled tumor cells were co-cultured with T cells at a 1:10 ratio (tumor: T cells) for 24 h. Flow cytometry analysis with gating on CFSE-positive cells was performed, and data are expressed as mean ± SD of triplicate experiments. Statistical significance between groups is indicated (* *p* < 0.05; *** *p* < 0.001, ns: not significant).

PARP inhibitors not only promote ferroptosis, but also have immunomodulatory effects, mainly recruiting CD8^+^ T cells and activating antitumor immune effects in BRCA-mutated TNBC [25,38]. We explored the influence of PARP inhibitor-induced upregulation of NPC1L1 on the behavior of CD8^+^ T cells in breast cancer. Leveraging diverse algorithms from the Tumor Immune Estimation Resource (TIMER) database, we discovered an inverse relationship between NPC1L1 expression levels and the infiltration of CD8^+^ T cells in breast cancer tissues (Figure 6A). Moreover, correlation analysis from TIMER indicated a negative association between NPC1L1 expression and the expression of T cell activation markers, including CD8A, CD69, and GZMB (Figure 6B). In mRNA sequencing data from MDA-MB-231, a TNBC cell line treated with Niraparib for 48 h, the T cell receptor signaling pathway was notably among the top 20 enriched pathways for gene alternative splicing (Figure 6C). Further, employing the CIBERSORT algorithm from TIMER to assess patient survival, we observed that patients with low NPC1L1 expression coupled with high CD8^+^ T cell infiltration had a more favorable prognosis compared to those with high NPC1L1 expression and high CD8^+^ T cell infiltration (Figure 6D). This suggests that NPC1L1 inhibition might hold clinical promise in augmenting the activity of CD8^+^ T cells infiltrating breast cancer.

Cytotoxic CD8^+^ T cells are the preferred immune cells targeting cancer, which exert specific cytotoxic effects by binding to the antigen peptide-MHC class I complex on the surface of tumor cells through perforin/granzyme pathway, Fas/FasL pathway, and TNF-TNFR pathway [39]. Given the relatively pronounced upregulation of NPC1L1 by the PARP inhibitor Niraparib, we conducted a combination experiment with the NPC1L1 inhibitor Ezetimibe. This study was designed to further explore whether NPC1L1 inhibition could enhance T cell apoptosis induced by PARP inhibitors. Tumor cells were pre-treated with the drugs, labeled with carboxyfluorescein diacetate succinimidyl ester (CFSE) for 48 h, and then co-cultured with activated peripheral blood mononuclear cells (PBMCs) at effector-to-target ratios (1:10) for an additional 24 h. Flow cytometry analysis demonstrated that the apoptosis rate in two TNBC cell lines was significantly elevated when co-cultured with T cells, in comparison to the control group lacking T cells. (Figure 6E). Notably, in the experiments targeting MDA-MB-231 cells, the apoptosis rate in the combination treatment group co-cultured with T cells was threefold higher than in the group without T cells. Additionally, the co-culture experiment demonstrated that the apoptosis rate in the group treated with the combination of Niraparib and Ezetimibe was significantly higher than in the Niraparib-only group, by a factor of approximately 1.5. These findings suggest that the combination of a PARP inhibitor with an NPC1L1 inhibitor significantly enhances T cell-mediated tumor cell killing.

### 3.7. Evaluation of the Antitumor Activity of PARP Inhibitor Niraparib in Combination with AZD5363 or Ezetimibe in a 4T1 Tumor-Bearing Mouse Model

**Figure 7:**
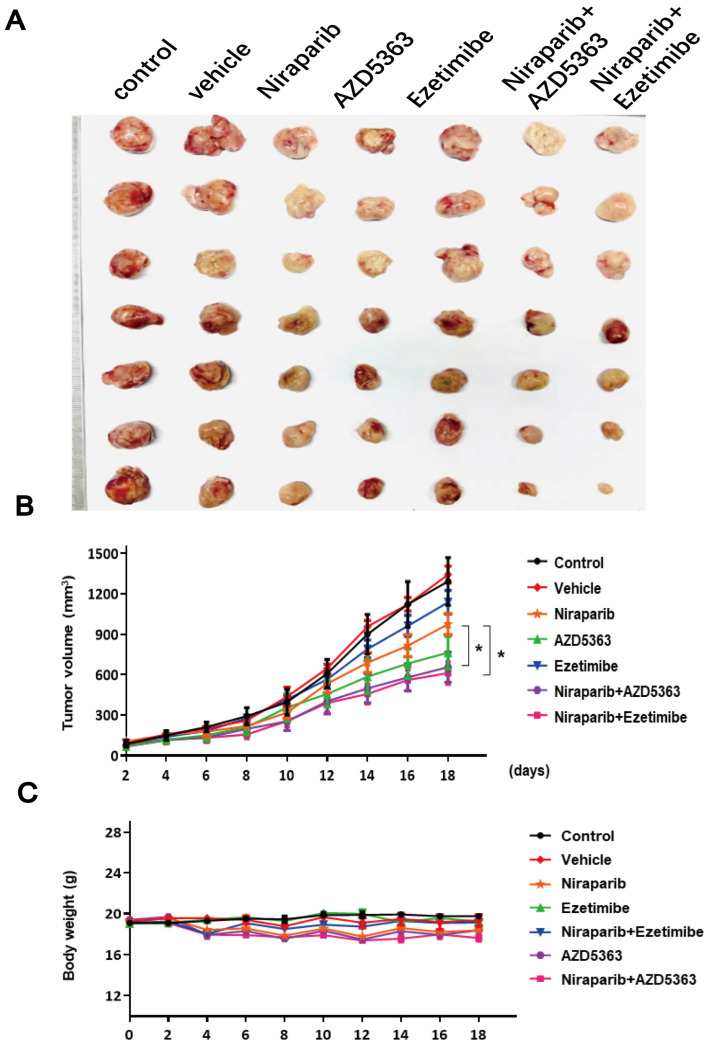
Antitumor efficacy of drug combinations in 4T1 tumor-bearing mouse models. (**A**) Representative image of tumors from each group of mice (*n* = 7). (**B**) Tumor growth curves of 7 groups of mice treated with drug combinations (25 mg/kg for Niraparib, 8 mg/kg for AZD5363, and 50 mg/kg for Ezetimibe) for 18 days. (**C**) Body weight of mice for 18 days observation. Statistical significance between groups is indicated (* *p* < 0.05).

Based on the remarkable effect of PARP inhibitors in combination with AZD5363 or Ezetimibe in inhibiting TNBC cell lines, we finally tested the antitumor effect of different drug combinations in a 4T1 tumor-bearing mouse model. Mice were given 25 mg/kg of Niraparib in combination with 8 mg/kg of AZD5363 or 50 mg/kg of Ezetimibe by intraperitoneal injection for 18 days. These doses, route, and frequency of compound administration were chosen because they were well tolerated and not lethal. The two drug combinations had a potently inhibitory effect on tumor growth, and they appeared to have similar tumor suppressive effects (Figure 7A,B). In addition, there was no significant weight loss during treatment in the drug combination group compared with the Niraparib group, suggesting less toxic side effects of PARP inhibitors in combination with AZD5363 or Ezetimibe (Figure 7C). The difference is that the combination of Niraparib and Ezetimibe appears to be safer than the combination of Niraparib and AZD5363. In conclusion, from a lipid regulation perspective, this study demonstrated the synergistic effect of a PARP inhibitor and AZD5363 or Ezetimibe.

### 3.8. The Combined Use of PARP Inhibitors and NPC1L1 Inhibitors Activates the In Vivo Immune-Mediated Tumor-Killing Effect

**Figure 8:**
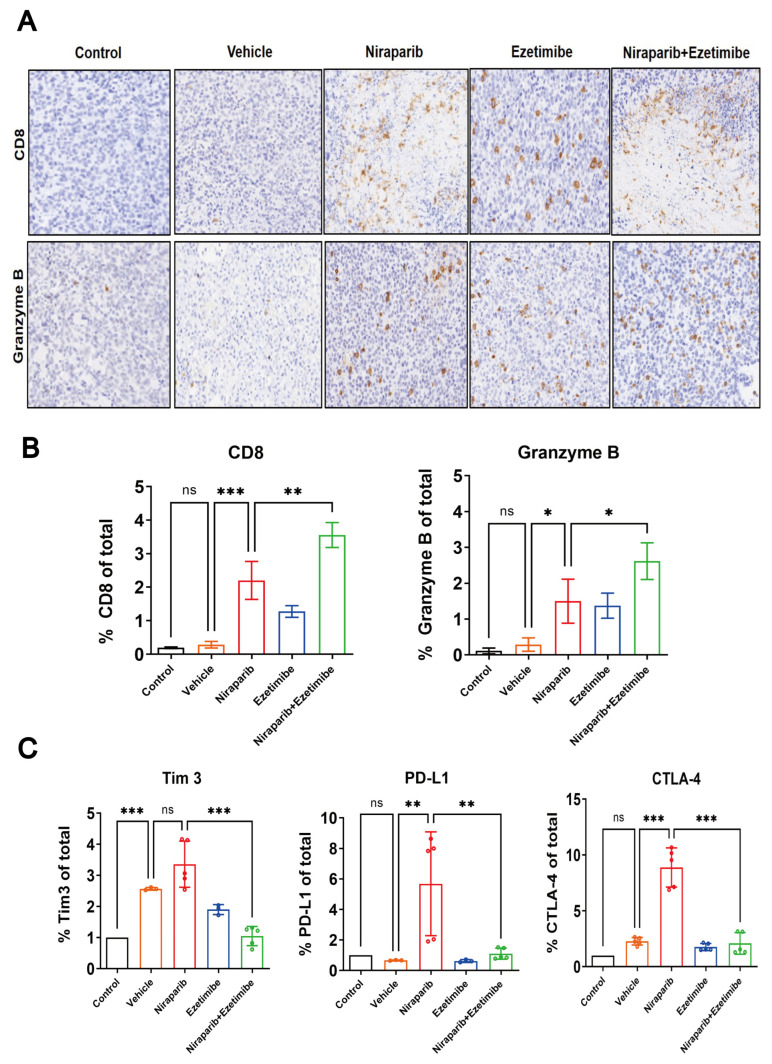
The combined use of PARP inhibitors and NPC1L1 inhibitors activates the in vivo immune-mediated tumor-killing effect. (**A**) The representative IHC staining images of protein CD8 and Granzyme B on tumor tissues. (**B**) Quantitative analysis of the contents of CD8 and Granzyme B in each group from immunohistochemical data using ImageJ software. (**C**) The mRNA levels of immune checkpoint molecules in tumor tissues were detected by RT-qPCR. Statistical significance between groups is indicated (* *p* < 0.05; ** *p* < 0.01; *** *p* < 0.001, ns: not significant).

To further validate whether the combination of Niraparib and Ezetimibe increases the infiltration of CD8^+^ T cells in the tumor tissue of immunocompetent mice, we conducted immunohistochemical experiments on dissected tumor tissues. The excised tumor tissues were fixed in 4% paraformaldehyde, dehydrated, embedded, and sectioned. They were then incubated overnight with CD8 antibody and Granzyme B antibody. Subsequently, after restaining, dehydration, and mounting, images were captured under a microscope, and the expression of CD8 and Granzyme B in the tumor tissues of each group was analyzed using ImageJ software. Both the Niraparib and Ezetimibe monotherapy groups promoted the infiltration of CD8^+^ T cells and increased the secretion of Granzyme B in the tumor tissue. Furthermore, the combination therapy group showed a further increase in the infiltration of CD8^+^ T cells and the expression of Granzyme B in the tumor tissue compared to the monotherapy groups (Figure 8A,B). This indicates that the combination of Niraparib and Ezetimibe inhibits tumor growth by increasing the infiltration of CD8^+^ T cells in the tumor tissue, and the increased expression of Granzyme B suggests an enhanced cytolytic effect of the combination therapy on CD8^+^ T cells.

Niraparib and Ezetimibe combination therapy has a significant antitumor effect, and we want to explore the impact of combination therapy on the immune microenvironment in tumor tissues. Tumor tissue blocks from mice after treatment were collected and thoroughly ground, and the changes in the expression of immune checkpoint molecules were detected. TIM-3 (T cell immunoglobulin domain and mucin domain-3), PD-L1 (Programmed death-ligand 1), and CTLA-4 (Cytotoxic T-lymphocyte-associated protein 4) are immune checkpoint molecules that play important roles in regulating the immune system, particularly in tumor immunity. TIM-3 is an inhibitory receptor primarily expressed on activated T cells, involved in regulating T cell activity and promoting immune tolerance. The expression of TIM-3 is associated with T cell exhaustion and tumor immune evasion [40]. PD-L1 is a protein mainly expressed on antigen-presenting cells. It binds to the PD-1 receptor on T cells, inhibiting T cell activation and proliferation, thus maintaining immune tolerance. Studies have shown that the expression level of PD-L1 in TNBC tissue is significantly higher than that in non-TNBC tissue [41]. CTLA-4 is another inhibitory receptor on T cells, competing with CD28 to bind to B7 molecules (such as CD80 and CD86), thereby inhibiting T cell activation. Blocking CTLA-4 can enhance the immune response of T cells to tumors. A multicenter, phase II study evaluated the efficacy and safety of the anti-PD-L1/CTLA-4 bispecific antibody KN046 in combination with nab-paclitaxel as first-line treatment for metastatic TNBC, demonstrating good tolerability and therapeutic effect [42]. Therefore, based on the results of our experiments, it can be determined that the single-agent Niraparib group significantly upregulated their expression, while the combination group of Niraparib and Ezetimibe reversed this upregulation (Figure 8C). In summary, Niraparib and Ezetimibe combined therapy can alter the immune microenvironment by regulating the expression of immune checkpoint molecules, enhance the invasion and cytotoxicity of tumor tissues, and play a significant antitumor role. This provides a new strategy and prospect for immunotherapy of TNBC.

## 4. Discussion

PARP inhibitors have revolutionized the treatment of BRCA-mutated breast and ovarian cancers by exploiting synthetic lethality in HRD tumors. However, many patients still do not benefit from these drugs. To address this, emerging strategies are shifting beyond the DNA repair-centric paradigm to target vulnerabilities linked to PARP’s non-canonical roles.

An increasing number of studies have shown that lipid metabolism induces resistance in tumor cells by affecting their behavior in the later stages of treatment [43]. When it comes to breast cancer, cholesterol has become a risk factor for developing the disease and is closely linked to its prognosis [44]. Our data indicate that PARP inhibitors cause a reprogramming of lipid metabolism, with a significant upregulation of NPC1L1 in this change. Preclinical and epidemiological studies have also suggested a significant correlation between inhibiting NPC1L1 and a reduction in breast cancer incidence [21]. Using the bc-GenExMiner v5.1, we evaluated the relationship between prognostic and NPC1L1 gene expression in TNBC patients. The analysis of patient survival using the Kaplan–Meier method showed that high expression of the NPC1L1 gene was associated with poorer prognosis in TNBC patients.

Currently, research on NPC1L1’s role in breast cancer is sparse, and the precise mechanism by which PARP inhibitors regulate NPC1L1 in TNBC remains uncharted. Our findings reveal that PARP inhibitors upregulate the expression of NPC1L1 through the RELA transcription factor, which in turn activates the AKT pathway. Therapeutically, targeting NPC1L1 with Ezetimibe synergized with Niraparib to suppress TNBC proliferation. Combination indices and reduced IC_50_ values across cell lines validated the synergy, likely attributable to dual disruption of cholesterol metabolism and AKT signaling. Similarly, combining Niraparib with the AKT inhibitor AZD5363 amplified cytotoxicity. Notably, both combinations potentiated ferroptosis, as evidenced by glutathione depletion and elevated lipid peroxidation. Cholesterol accumulation via NPC1L1 may initially buffer membrane fluidity, but its inhibition redirects lipid metabolism toward PUFA-enriched membranes, priming cells for ROS-driven ferroptosis. Consequently, the targeted inhibition of NPC1L1 in conjunction with PARP inhibitors presents a novel therapeutic combination for the treatment of TNBC.

Statins, known for their cholesterol-lowering properties, have garnered attention for their potential effects in cancer therapy, with a particular preference for TNBC as indicated by research findings [45,46,47]. Despite their potential to curb the progression of breast cancer, the current body of observational research does not strongly advocate for the advancement of adjuvant statin therapy into clinical trials [48]. On a related note, Ezetimibe, an FDA-approved lipid-lowering drug, has demonstrated promise in the treatment of TNBC [22]. Our tests have confirmed that Ezetimibe, an NPC1L1 inhibitor, exhibits significant inhibitory activity against various breast cancer cell lines. Notably, in combination therapy models, the pairing of Ezetimibe with the PARP inhibitor Niraparib has yielded a markedly synergistic lethal effect on TNBC cells. Mendelian randomization simulations provide experimental observations suggesting that the long-term use of Ezetimibe is not associated with an increased risk of cancer [49]. This evidence bolsters the potential of Ezetimibe as a supportive medicine for cancer treatment and prevention in the future.

According to reports, different subtypes of TNBC have mutations/alterations in the PI3K/AKT pathway. AKT mutations can lead to chemotherapy resistance, and AKT has been proven to be an effective therapeutic target for TNBC. AKT inhibitors such as Ipatasertib, Uprosertib, and AZD5363 have entered clinical trials for treating TNBC [50]. Considering our findings in transcriptomics that Niraparib can activate the AKT pathway in TNBC cells, we have combined the novel AKT inhibitor (AZD5363) with Niraparib for the treatment of TNBC. This combination has been shown to effectively sensitize the killing effect of PARP inhibitors in vitro and in vivo, proving that this combination therapy is also a potential new treatment strategy.

Studies have shown that the synthesis and intake of cholesterol have an impact on cancer progression and also play a role in tumor biology processes, such as ferroptosis [51]. Ferroptosis is a form of iron-dependent cell death caused by uncontrolled lipid peroxidation. GSH is a major reducing substrate and is essential for the prevention of ferroptosis [52]. The relationship between cholesterol and ferroptosis, a form of regulated cell death dependent on iron and characterized by lipid peroxidation, can be multifaceted. Cholesterol is a vital component of the cell membrane, playing a crucial role in maintaining its fluidity and integrity. As ferroptosis is associated with the peroxidation of membrane lipids, cholesterol’s influence on the cell membrane’s properties could indirectly affect the susceptibility to ferroptosis [13]. In terms of pharmacological intervention, certain cholesterol-lowering drugs, such as statins and Ezetimibe, could indirectly affect the process of ferroptosis by modulating cholesterol metabolism. These drugs might possess the potential to counteract ferroptosis by reducing cholesterol levels and, consequently, the incidence of lipid peroxidation in the cell membrane [53]. We found that the synergistic effect of Niraparib combined with Ezetimibe or AZD5363 is achieved by inhibiting cholesterol metabolism, thereby making breast cancer cells more susceptible to ferroptosis.

The reprogramming of lipid metabolism not only activates relevant signaling pathways to promote the proliferation and metastasis of tumor cells [9,54], but also affects the immune response of tumors [55]. A team has found that treatment of prostate cancer patients with NPC1L1 inhibitor Ezetimibe promoted the infiltration of CD8^+^ T cells into prostate tumors [56]. When two TNBC breast cancer cell lines with BRCA mutations were co-cultured with T cells, the apoptosis rate in the combination group of PARP inhibitor Niraparib and NPC1L1 inhibitor Ezetimibe was 1.5 times that of the Niraparib monotherapy group, indicating that inhibition of NPC1L1 enhances the immune effect of PARP inhibitors.

After receiving treatment with PARP inhibitors, tumor cells release double-stranded DNA fragments, thereby activating the stimulator of interferon genes (STING) signaling pathway in dendritic cells within the tumor, triggering type I interferon (IFN) response to recruit CD8^+^ T cells [25]. However, in BRCA wild-type tumors, the DNA damage induced by PARP inhibitors and the STING pathway-triggered type I IFN response are limited [38]. As a single agent, Niraparib exerts minimal influence on the infiltration of CD8^+^ T cells, marked by CD3 and CD8, as well as their activity, indicated by Granzyme B levels. This indicates that the immunomodulatory function of Niraparib is not significantly associated with effective antitumor effects in the in vivo model of BRCA wild-type breast cancer. Therefore, an increasing number of researchers are developing various combination therapy regimens to enhance the efficacy of PARP inhibitors. NPC1L1 controls the transport of cholesterol, which not only directly affects the structure of PD-L1 but also influences the activity of PD-L1 through protein pathways [57,58]. PARP inhibitors mediate the upregulation of PD-L1, which weakens its immunotherapeutic effect, and NPC1L1 may have a potential connection with it. Targeting NPC1L1 can reduce cholesterol, thereby reducing the upregulation of PD-L1 and enhancing the immunotherapeutic effect of PARP inhibitors. This study provides two novel combination therapies of PARP inhibitors for the treatment of TNBC, providing sufficient basis for future clinical researches.

In our study, we found that PARP inhibitors upregulate the expression of NPC1L1 by modulating the RELA transcription factor, thereby triggering the activation of the downstream AKT signaling pathway. The combination of Niraparib with Ezetimibe (a cholesterol transport inhibitor) effectively inhibited tumor growth in an immunocompetent mouse 4T1 model without significant toxic side effects. It also promoted the infiltration of CD8^+^ T cells, facilitated the secretion of granzyme B by these cells, and significantly reduced the expression of immune checkpoints. These findings provide valuable insights into lipid metabolism and its interactions with immune regulation in TNBC, presenting a novel therapeutic strategy that leverages the combined use of PARP inhibitors for the treatment of TNBC.

## 5. Conclusions

This study elucidates the molecular mechanism by which PARP inhibitors drive lipid metabolic reprogramming via the NPC1L1-AKT axis in TNBC. We demonstrate that combinatorial targeting of cholesterol metabolism (e.g., NPC1L1 inhibition) or AKT signaling synergistically enhances ferroptosis induction and potentiates antitumor immunity. Key findings highlight that cholesterol metabolism serves as a critical vulnerability in TNBC, with NPC1L1 inhibition augmenting CD8^+^ T cell infiltration and cytotoxicity (Graphical Abstract). These results not only deepen the understanding of PARP inhibitors-mediated therapeutic effects but also propose a novel “metabolic-immune” dual-targeting strategy for precision treatment of TNBC.

## Figures and Tables

**Table 1:** Primers used for fluorescence real-time quantitative PCR experiments are as follows.

Gene	Primer Direction	Sequence (5′→3′)
PARP1-(homo)	Forward	CTTGACAACCTGCTGGACAT
	Reverse	CCTGATGATCTCGGCTTCTTC
NPC1L1-(homo)	Forward	GAGCTGCATGGCTGACTACG
	Reverse	CCGCAGGGTAATTGTTGAG

**Table 2:** siRNA sequences (Genomeditech, Shanghai, China) were utilized.

Gene	Primer Direction	Sequence (5′→3′)
PARP1-Homo-1	sense	GGAUGGGUUCUCUGAGCUUTT
	antisense	AAGCUCAGAGAACCCAUCCTT
PARP1-Homo-2	sense	GAGGAAGGUAUCAACAAAUTT
	antisense	AUUUGUUGAUACCUUCCUCTT
NPC1L1-Homo	sense	GAGGCCUUCUUAGAGGAAATT
	antisense	UUUCCUCUAAGAAGGCCUCTT

**Table 3:** Two independent shRNA sequences (Sangon Biotech, Shanghai, China) were used.

Gene	Primer Direction	Sequence (5′→3′)
RELA (103374-1)	sense	GATTGAGGAGAAACGTAAA
	antisense	TTTACGTTTCTCCTCAATC
RELA (103375-1)	sense	CTTAATAGTAGGGTAAGTT
	antisense	AACTTACCCTACTATTAAG

**Table 4:** The IC_50_ of single-agent and two-drug combination treatments in the TNBC cell lines.

Cell Lines	Niraparib (μM)	Ezetimibe (μM)	AZD5363 (μM)	Niraparib (μM) + Ezetimibe (μM)	Niraparib (μM) + AZD5363 (μM)
MDA-MB-231	20.52	31.76	14.26	9.18/13.77	7.84/11.76
MDA-MB-453	30.56	44.34	10.28	18.76/18.76	7.27/10.90
MDA-MB-468	23.79	38.03	10.57	12.36/18.54	2.04/3.06
HCC1937	49.37	23.12	12.79	7.42/7.42	7.73/11.59

## Data Availability

All data and associated protocols are included in the manuscript and available to the readers. Cell lines generated in this study are available upon request.

## Abbreviations

poly (ADP-ribose) polymerase PARP	PARP
triple-negative breast cancer	TNBC
polyunsaturated fatty acid	PUFA
homologous recombination deficiency	HRD
7-dehydrocholesterol	7-DHC
7-dehydrocholesterol reductase	DHCR7
Niemann-Pick C1-Like 1	NPC1L1
tumor microenvironment	TME
lipid peroxidation	LPO
methyl tert-butyl ether	MTBE
glutathione	GSH
oxidized glutathione	GSSG
5,5′-dithiobis (2-nitrobenzoic acid)	DTNB
1-methyl-2-vinylpyridinium triflate	M2VP
thiobarbituric acid	TBA
malondialdehyde	MDA
phosphate-buffered saline	PBS
3-(4,5-dimethylthiazol-2-yl)-2,5-di-phenyltetrazolium bromide	MTT
peripheral blood mononuclear cells	PBMCs
fetal bovine serum	FBS
carboxyfluorescein succinimidyl ester	CFSE
Principal component analysis	PCA
partial least squares-discriminant analysis	PLS-DA
fold change	FC
phosphorylated AKT	*p*-AKT
Combination Index	CI
phosphatidylcholine	PC
phosphatidylethanolamine	PE
coding sequence	CDS
highest single agent	HSA
Tumor Immune Estimation Resource	TIMER
carboxyfluorescein diacetate succinimidyl ester	CFSE
reactive oxygen species	ROS
fold change	FC
cholesterol esters	ChE
endosomal recycling compartment	ERC
T cell immunoglobulin domain and mucin domain-3	TIM-3
Programmed death-ligand 1	PD-L1
Cytotoxic T-lymphocyte-associated protein 4	CTLA-4
stimulator of interferon genes	STING
interferon	IFN
